# Endosperm cell death: roles and regulation in angiosperms

**DOI:** 10.1093/jxb/erae052

**Published:** 2024-02-14

**Authors:** Nicolas M. Doll, Moritz K. Nowack

**Affiliations:** 1 Department of Plant Biotechnology and Bioinformatics, Ghent University, Ghent 9052, Belgium; 2 VIB Center of Plant Systems Biology, Ghent 9052, Belgium

**Keywords:** Angiosperm, cell death, cell elimination, endosperm, programmed cell death, PCD, seed development, germination

## Abstract

Double fertilization in angiosperms results in the formation of a second zygote, the fertilized endosperm. Unlike its embryo sibling, the endosperm is a transient structure that eventually undergoes developmentally controlled programmed cell death (PCD) at specific time points of seed development or germination. The nature of endosperm PCD exhibits a considerable diversity, both across different angiosperm taxa and within distinct endosperm tissues. In endosperm-less species, PCD might cause central cell degeneration as a mechanism preventing the formation of a fertilized endosperm. In most other angiosperms, embryo growth necessitates the elimination of surrounding endosperm cells. Nevertheless, complete elimination of the endosperm is rare, and in most cases, specific endosperm tissues persist. In mature seeds, these persisting cells may be dead, such as the starchy endosperm in cereals, or remain alive to die only during germination, like the cereal aleurone or the endosperm of castor beans. In this review, we explore the current knowledge surrounding the cellular, molecular, and genetic aspects of endosperm PCD, and the influence environmental stresses have on PCD processes. Overall, this review provides an exhaustive overview of endosperm PCD processes in angiosperms, shedding light on its diverse mechanisms and its significance in seed development and seedling establishment.

## I) Introduction

The emergence of flowering plants was associated with a drastic change in the mode of sexual plant reproduction. In angiosperms, highly specialized reproductive organs form the flower in which meiosis takes place and strongly reduced gametophytes develop. Gametes are brought together in the archetypical double fertilization process that produces not one, but two zygotic structures: Next to the fertilized egg cell that develops into the embryo, the female gametophytic central cell gets fertilized to form the nutritive tissue of the endosperm. Making the onset of nutrient delivery to the seed dependent on fertilization provided the advantage of focusing maternal investments into successfully fertilized seeds (Baroux et al., 2002). Together with the evolution of a plethora of different flower morphologies and pollination strategies, double fertilization and has been interpreted as a major reason for the evolutionary success and diversification of angiosperms (Friedman, 1998).

Consequently, the vast majority of angiosperm seeds contains two zygotic structures, the embryo and the surrounding endosperm. Both embryo and endosperm develop alongside each other encapsulated by the maternal tissues of the seed coat, but they have radically diverging developmental fates: Embryo development will generate a new organism with embryonic plant organs, while the endosperm develops into a highly specialized nutritive organ. This difference is already predetermined by the largely divergent female gametes: the egg is a small cell close to the micropyle, while the large, central cell, most commonly homodiploid, represents the bulk of the female gametophyte. Directly after fertilization, the endosperm usually undergoes a fast expansion supported by coenocytic free nuclear divisions to generate a nutrient sink ensuring its function of nutritive tissue (Dumas and Rogowsky, 2008; Raghavan, 2003).

Endosperm development is highly variable among angiosperms. In species of the Orchidaceae and Podostemaceae families, endosperm development is suppressed, while in cereals the endosperm is the largest seed tissue undergoing a complex development and differentiates into several distinct tissues with different functions (Johri, 1984). However, in most angiosperms, the endosperm has a key nutritive role, either in transferring nutrients from the maternal tissues towards the embryo and/or in storing a part of these nutrients to fuel the seedling growth during germination. In some taxa like cereals, an enormous amount of reserves is stored in endosperm cells, making the endosperm a paramount tissue for humans as a source of food, feed and energy (Olsen, 2001; Sabelli and Larkins, 2009).

Unlike the embryo that develops into an adult plant after seed development, the endosperm terminates its development and finally dies during seed development or germination. Endosperm death has been suggested to be the result of different forms of genetically regulated programmed cell death (PCD) (Van Hautegem et al., 2015; Young and Gallie, 2000a). Developmentally regulated PCD (dPCD) is a tightly regulated process that is crucial during the development and the growth of a plant (Daneva et al., 2016; Locato and De Gara, 2018). Though sharing several morphological and molecular characteristics (Hofmann, 2020), plant genomes do not encode clear orthologs of key PCD regulators in animal systems (Minina et al., 2021). Nevertheless, forms of dPCD, as well as PCD types triggered by external biotic and abiotic factors, are crucial processes in plant development, reproduction, stress adaptation, and immunity (Daneva et al., 2016; Maekawa et al., 2023; Paes de Melo et al., 2022).

While all endosperm cells eventually perish, there is a great diversity in the timing and mode of cell death execution in the different endosperm tissues of the diverse angiosperm species. The molecular regulation of the different manifestations of endosperm PCD remains little understood. However, recent studies have started to increase our understanding of the molecular genetic pathways of some endosperm dPCD processes. In this review we will discuss the different dPCD processes occurring in the endosperm of angiosperms, which we have divided into four main categories: i) death of the central cell to avoid endosperm development, ii) elimination of endosperm cells around the embryo to facilitate the invasive growth of the embryo during seed development, iii) the cell death process in the starchy endosperm of cereals terminating seed filling, iv) the germination-induced death of endosperm tissues persisting throughout seed development. In the following, we will highlight the current knowledge of cellular processes occurring during cell death and their molecular control. Finally, we will also discuss the often still hypothetical functions of dPCD in these different contexts.

### I) Cell death as a way to avoid endosperm formation

The development of a fertilized endosperm has been described as a synapomorphy of the angiosperm clade, and seeing its prevalence, it appears to be of evolutionary advantage for most angiosperm species. However, under particular selection pressures, developing an endosperm may be disadvantageous. Some species may draw benefits from a reduction of seed size, often alongside an increase in seed numbers produced per fruit.

Many species of the *Podostemaceae*, a family of aquatic plants, are characterized by a strongly reduced seed size, and the absence of a developed endosperm (Rutishauser, 1997). Possibly, in aquatic environments there are less benefits from large seeds that may promote seed survival, longevity, and seedling establishment in terrestrial habitats. Conversely, producing numerous small seeds might be of advantage in aquatic environments, for instance by increasing their dispersal potential and by facilitating their adherence to the small cracks of the solid underwater surfaces prior to germination (M Leleeka Devi et al., 2016; Philbrick and Alejandro Novelo, 1997). Endosperm development can be suppressed by the specific death of the gametophytic endosperm progenitor cell, the central cell, before or directly after fertilization. In several species of the *Podostemaceae*, the usually large central cell is strongly reduced in size and degenerates before the pollen tube reaches the ovule (Sehgal et al., 2014, 2011). In *Marathrum schiedeanum*, the degenerating central cell presents features of PCD, including chromatin condensation, shrinkage of cytoplasm and nucleus, and appearance of autophagic vacuoles (Jiménez-Durán et al., 2021). The regulation of central cell dPCD in *Podostemaceae* is still poorly understood but the close correlation between pollination and central cell death suggests a long-distance signal from the pollen that triggers central cell dPCD (Sehgal et al., 2014). However, this is not observed in all *Podostemaceae*; in *Marathrum schiedeanum* cell death happens before pollination, suggesting a different kind of regulation (Jiménez-Durán et al., 2021). Central cell dPCD might have been actively selected throughout evolution to promote the reduction of seed size. Possibly central cell dPCD may represent a case of developmental heterochrony, by bringing forward dPCD as the latest step of endosperm differentiation to a time point before fertilization.

Negative selection on seed size also happens in Orchids. Seeds of some species are so strongly reduced in size that they have been termed “dust seeds”. Orchid dust seeds can weigh as little as 0.3 μg with lengths down to 50 μm. Making small seeds allows the plant to produce a lot of seeds (up to 4 million seeds per fruit), for a rather reduced parental investment, optimizing thus seed dispersion. In mycoheterotrophic species, for which seedling establishment depends on the interaction with a symbiotic fungus, this is particularly important to maximize the chance of finding a symbiont. (Arditti and Ghani, 2000; Eriksson and Kainulainen, 2011). Within the orchid dust seed, the endosperm is often strongly reduced and, in some species, even fails to develop altogether. More investigations are needed to assess how widespread endosperm-less are in the orchids, and which mechanisms underlie the failure of endosperm development (Kodahl et al., 2015; Yeung, 2017). In *Spiranthes sinensis* for instance, double fertilization fails because of impaired sperm cell mitosis (Terasaka et al., 1979) while in *Listera ovata* and *Ophrys insectifera*, the male gametes are unable to reach the central cell (Arditti, 1992; Johri et al., 1992). Whether the absence of endosperm development shown in some orchid species involves an active PCD of the central cell prior to fertilization, as in the *Podostemaceae*, or of the young endosperm, is still unclear and should be investigated in the future.

## II) Cell elimination in the embryo-adjacent endosperm

### 1) Endosperm elimination and impact on embryo endosperm ratio in mature seed

In contrast to the situation in many Orchids and the *Podostemaceae*, the fertilized central cell in most angiosperms develops into an expansive endosperm composed of one or more distinct tissues. Usually, the early endosperm proliferates much faster than the embryo. This may have several reasons, including the large volume of the fertilized central cell, the polyploidy of the endosperm, and the fact that early endosperm proliferation often occurs in a free-nuclear coenocytic fashion that can be accomplished faster than the precisely executed cytokineses that have to establish the complex body plan in the embryo (Boisnard-Lorig et al., 2001; Dumas and Rogowsky, 2008; Mansfield and Briarty, 1991). Due to its fast proliferation, the early endosperm acts as a sink tissue that acquires and stores nutrients from the mother plant. Hence, this early stage of endosperm is a crucial determinant for the size and nutrient storage potential of the mature seed (Ingram, 2020).

**Figure 1:**
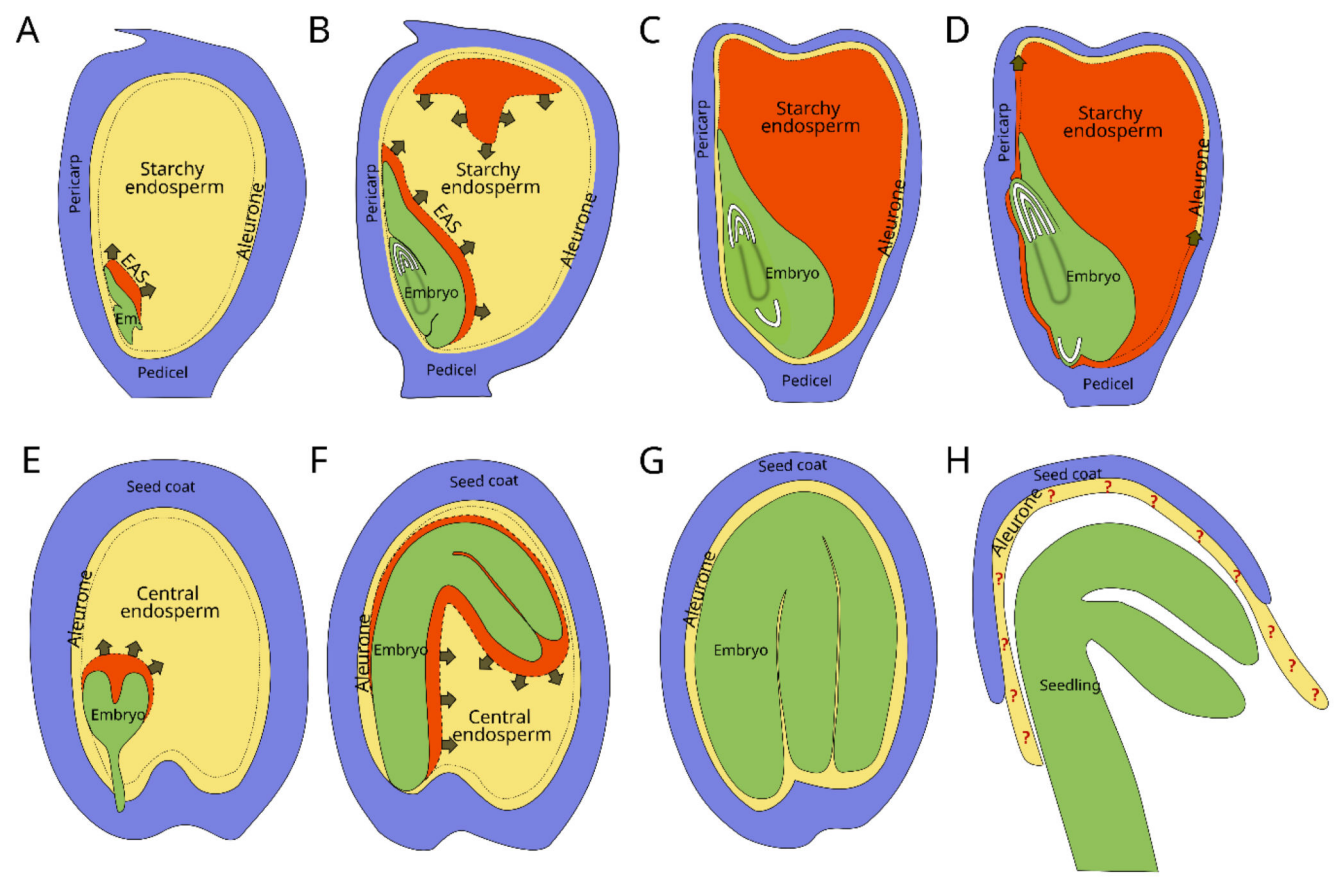
The various endosperm tissues in maize and Arabidopsis undergo PCD at different developmental stages. Endosperm tissues undergoing cell death are indicated in red. Arrows indicate the direction of cell death progression. A-D) Maize kernels at different developmental stages. Arrows indicate cell death progression. A) At 11 DAP, the endosperm adjacent to the scutellum (EAS) undergoes cell death and are eliminated, making space for the growing embryo. B) At 19 DAP, EAS cell death continues, and a conservative cell death process is occurring in the central crown and the center of the starchy endosperm. C) In mature kernels, the entire starchy endosperm is dead but the aleurone layer remains alive. D) During germination, the aleurone cells undergo PCD. The cells close to the embryo die first. E-H) Arabidopsis seeds at different developmental stages. E) At early torpedo stage, the embryo begins its invasive growth of the endosperm. The endosperm cells close to the embryo undergo PCD and are eliminated. F) This process continues and amplifies at the bent cotyledon stage. G) At maturity, the entire central endosperm has been eliminated and only a single layer of aleurone cells remains present and alive. H) During germination, the aleurone undergoes a process of vacuolization. It is unclear, however, whether aleurone cells finally undergo a PCD process or not.

The initially slow growth of the embryo picks up speed in mid-seed development. As the space available within a seed is limited by the confines of the seed coat, embryo expansion occurs as an endosperm-invasive growth that is associated with the elimination of the surrounding endosperm (Doll et al., 2023; Marsollier and Ingram, 2018). Elimination of endosperm cells determines the degree of embryo expansion and hence the proportion of endosperm that remains in the mature seed. In non-endospermic species such as in *Vicia faba* and *Phaseolus coccineus (Fabaceae)*, or *Echinocystis lobate (Cucurbitaceae)*, the entire endosperm is eliminated during seed development and maturation (Johansson and Walles, 1994; Lombardi et al., 2007; Wojciechowska and Olszewska, 2003). By contrast, in some endospermic species including cereals or Euphorbiaceae, a large part of the endosperm remains present in the mature seed (Domínguez and Cejudo, 2014; Sabelli, 2012; Schmid et al., 1999) (Figure 1). In others, only a small portion of the endosperm remains. In quinoa, the micropylar region persists (M. P. López-Fernández and Maldonado, 2013), while in the model species Arabidopsis (*Arabidopsis thaliana)* the entire outer cell layer of the peripheral endosperm remains present in mature seeds (Brown et al., 1999). Similar to the outermost endosperm layer(s) in cereal seeds, the aleurone, this outer endosperm layer in Arabidopsis remains viable until after seed germination, and has hence been considered aleurone-like by some authors (Bethke et al., 2007).

To date is remains unclear how the degree of endosperm elimination is determined. Mutants in maize or rice with enhanced embryo growth show a proportionally smaller endosperm, indicating that gradual embryo expansion and endosperm elimination are coordinated (Yang et al., 2013; Zhang et al., 2012). However, which signals achieve this coordination, and how the surviving layer of peripheral endosperm resists elimination is little understood to date.

### 2) Cytological features of the execution of endosperm elimination

**Figure 2:**
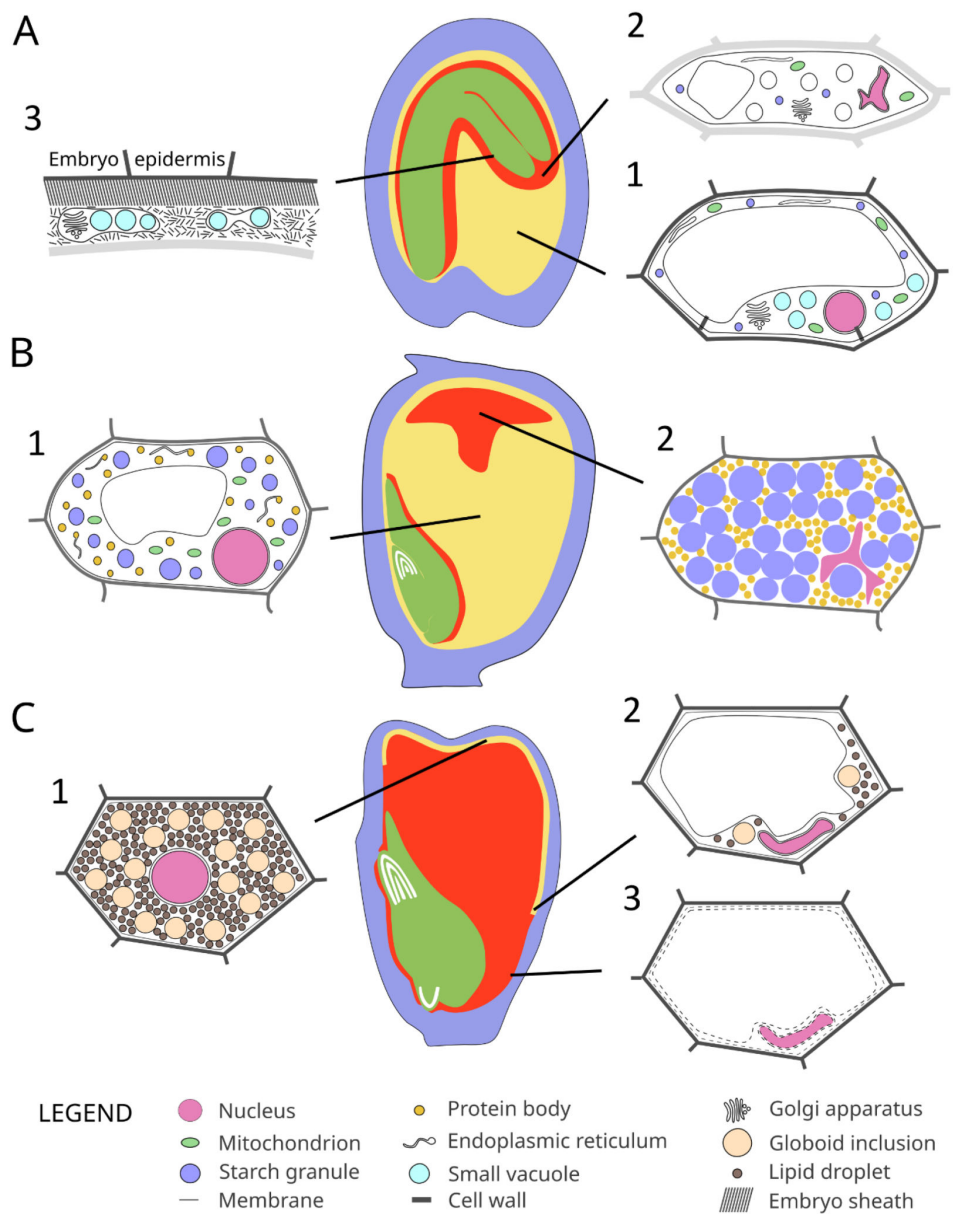
Subcellular features of PCD execution in different endosperm tissues. Dead or dying tissues are indicated in red. A) Scheme of an Arabidopsis seed at the bent-cotyledon stage. A central endosperm cell before PCD is represented (1). During PCD, the vacuole and the cell shrinks, the cell walls are remodeled and the nucleus collapses (2). After cellular breakup, the remaining organelles, vesicles, and membrane systems are gradually degraded. Cell corpses are processed into a matrix of fibrous material that is visually similar to the highly organized embryo sheath that covers the embryonic epidermis (3). B) Scheme of maize kernel at 19 days after pollination. Starchy endosperm cell before (1) and after (2) cell death. Note that starch granules, protein bodies and the cell walls are preserved after starchy endosperm PCD. The nucleus has collapsed and the genomic DNA has fragmented but nuclear remnants persist. C) Scheme of a maize kernel during germination. An aleurone cell filled with lipid droplets and protein storage vacuoles before germination is represented (1). During germination, but before PCD, the small vacuoles fuse into a large lytic vacuole, the lipid reserves are remobilized and the nucleus deforms (2). Eventually, aleurone cells lose vacuolar and plasma membrane integrity and die (3).

Due to its situation inside the developing seed and fruit tissues, analyzing the subcellular features of endosperm elimination is particularly challenging and requires analyses of sections of fixed material. In Arabidopsis, endosperm breakdown occurs in the close vicinity to the embryo and involves the progressive degradation of the cell wall, a shrinking of the central vacuole and the deformation and fragmentation of the nucleus. The endosperm cell eventually dies when the plasma membrane breaks down and cellular integrity is lost, though intriguingly at this stage many organelles appear to be still intact. The last phase of endosperm breakdown happens in this “cell-free” zone directly at the embryo interface. Here, all the cellular remains are cleared with the exception of a fibrous matrix that is visually similar to the embryo sheath (Doll et al., 2023) (Figure 2C-E). The embryo sheath is a well-organized structure surrounding the embryo that physically separates it from the surrounding endosperm during seed development and germination (Doll et al., 2020a; Moussu et al., 2017). The embryo sheath can be detected by anti-extensin antibodies, which also outlines the remains of degrading endosperm cells (Moussu et al., 2017). As extensins are known to be resistant to proteases and are able to self-assemble in covalently crosslinked polymeric networks (Cannon et al., 2008; Chen et al., 2015), this fibrous matrix might be composed of extensins that resist post-mortem endosperm corpse clearance and are integrated into the embryo sheath. In other species, the degree of cell corpse clearance is variable. In maize, the cell walls of dead endosperm cells adjacent to the embryo scutellum are not entirely cleared and accumulate at the embryo surface, which indicates a different mechanism of cell corpse clearance (Doll et al., 2020b). In *Solanum nigrum*, endosperm elimination resembles Arabidopsis with the deposition of a fibrous matrix between embryo and endosperm (BRIGGS, 1996; Briggs, 1993). In *Vicia faba* and *Phaseolus coccineus*, the endosperm does not cellularize, yet as the embryo progressively invades the endosperm, nuclei of the large coenocyte display cell death features (Johansson and Walles, 1994; Lombardi et al., 2007), suggesting that endosperm breakdown occurs in an ordered pattern despite the absence of cellular organization.

### 3) Endosperm elimination is genetically controlled

The study of endosperm elimination in Arabidopsis has revealed first insight into its genetic control. The invasion of the endosperm by the embryo requires the bHLH transcription factors ZHOUPI (ZOU) and INDUCER OF CBF EXPRESSION 1 (ICE1), which interact in heterodimers to function (Denay et al., 2014; Yang et al., 2008). Absence of either ZOU or ICE1 results in endosperm with thickened cell walls that cannot be invaded by the growing embryo, which consequently remains dwarf (Denay et al., 2014; Fourquin et al., 2016; Yang et al., 2008). The ZOU/ICE1 module has been shown to regulate cell wall modifying enzymes that are thought to soften the cell walls, enabling the initiation of embryo invasive growth. However, neither *ZOU* nor *ICE1* overexpression trigger ectopic dPCD in Arabidopsis (Kanaoka et al., 2008; Yang et al., 2008) and the endosperm cells in *zou* ultimately undergo dPCD and break down, although too late to allow embryo expansion (Doll et al., 2023). These data suggest that ZOU and ICE1 are involved in the preparation or initiation, rather than in the execution of endosperm elimination. The execution of endosperm dPCD is orchestrated by redundantly acting NAC transcription factors, including KIRA1 (KIR1/ANAC074), ORESARA1 (ORE1/ANAC092), ANAC046 and ANAC087. Inhibition of the NAC dPCD pathway results in reduced pace of endosperm elimination, limited cell corpse clearance, and less voluminous embryos. Simultaneous inhibition of both the ZOU and the NAC endosperm breakdown pathways results in persisting central endosperm that remains viable and intact until desiccation late in the seed maturation process, much longer than in the *zou* mutant (Doll et al., 2023). These recent findings indicate that the ZOU and NAC pathways act at least partially in parallel, though the NAC pathway appears to be activated developmentally downstream of the ZOU pathway.

The discovery of an endosperm-expressed canonical dPCD pathway controlled by NAC TFs that act in established dPCD contexts (Cubría-Radío and Nowack, 2019) implicates that the endosperm expresses an endogenous killing program to provide space for the growing embryo. This notion stands in contrast to earlier hypotheses positing that elimination of the endosperm softened by ZOU activity is a physical embryo growth-driven destruction process (Fourquin et al., 2016; Marsollier and Ingram, 2018). The role of the embryo in promoting endosperm cell death remains controversial. On the one hand, the specific activation of a dPCD gene regulatory network in the embryo-adjacent ESR could imply a mechanical or chemical signal from the embryo that promotes dPCD (Doll et al., 2023). On the other hand, the reduction of embryo growth, or even its absence, does not appear suppress endosperm cell death (Fourquin et al., 2016; Xiong et al., 2021), suggesting a certain degree of autonomy in the execution of endosperm dPCD. Possibly, there is an endosperm-intrinsic dPCD program that gets fine-tuned by embryo-derived signals to coordinate endosperm breakdown with embryo growth in a precise spatial and temporal manner. In conclusion, endosperm elimination is a critical process to ensure the optimal growth of the embryo. As such, the rate and proportion of endosperm elimination might become a potential target trait in agriculture. The embryo and the endosperm store different kind of reserve compounds, for instance, the cereal embryo stores higher amounts of oil than the endosperm and an altered embryo/endosperm ratio is associated with elevated oil content (Moose et al., 2004; Nagasawa et al., 2013). Hence, modifying embryo/endosperm ratios may represent a future strategy to optimize crop seed quality.

## III) Conservative starchy endosperm cell death in cereals

### 1 Cell death terminates starchy endosperm filling

In cereals, only a minor part of the endosperm is eliminated and replaced by the growing embryo. As a consequence, the endosperm occupies most of the grain volume of mature cereal seeds. However, with the exception of the outermost aleurone endosperm layer(s), the mature endosperm is dead at maturity. Starting in mid-grain development, all the cells of the central (starchy) endosperm will die in a gradually advancing process (Figure 1). Prior to their death, starchy endosperm cells accumulate large amounts of nutrients that are stored as starch in amyloplastic starch granules, and as endoplasmic reticulum-derived protein bodies (Zheng and Wang, 2015). Endosperm cell filling is quickly followed by cell death and the tight association between these two processes suggests that they are closely coordinated. In maize for instance, the cells in the apical crown region of the starchy endosperm are the first to be filled with nutrients and are also the first to die (Langer et al., 2023; Young et al., 1997). In rice, the central endosperm region that contains the first completely filled endosperm cells displays a transcriptomic signature of cell death (Ishimaru et al., 2022). As endosperm filling is an active metabolic process requiring living cells, we can assume that cell death effectively terminates the filling procedure. However, despite the relevance of starchy endosperm filling for human nutrition, the regulation of the cell death process in cereals remains poorly understood.

### 2 The cytological features of cereal starchy endosperm cell death

During starchy endosperm filling, storage organelles including protein bodies, protein storage vacuoles, and starch granules accumulate large amounts of nutrients, expanding within the cytoplasm and progressively replacing the large central vacuole (Zheng and Wang, 2015). After a cell is completely filled, a unique cell death process happens that renders starchy endosperm cell lifeless while their cell walls, starch granules and protein bodies remain in place after cell death (Khoo and Wolf, 1970; Young et al., 1997). Nuclei are deformed by growing starch granules and eventually appear to collapse and fragment (Kobayashi et al., 2013), becoming non-functional in producing mRNAs (Wegel et al., 2005). The genomic DNA of starchy endosperm cells has been described to undergo laddering, i.e. to be cleaved into small fragments. Loaded on agarose gels, the DNA forms ladders of 180 base-pair fragments corresponding to the size of internucleosomal linker regions that might be cleaved by the action of nucleases (Young et al., 1997; Young and Gallie, 1999). However, the fact that 4',6-diamidino-2-phenylindole (DAPI) labels nuclear remnants in the endosperm suggest that the fragmented DNA is not entirely cleared during cell death (Kobayashi et al., 2013; Li et al., 2004) (Figure 2A-B). Labeling of dying endosperm cells by the non-membrane-permeable dye Evans Blue (Young et al., 1997; Young and Gallie, 1999) suggests that the plasma membrane loses integrity during cell death. A compromised membrane integrity has even been reported before the completion of cell filling (Golovina et al., 2000), however, how a cell with a compromised membrane should be able to maintain the energy-intensive process of seed filling is not understood. Regarding the organelles preserved during and after cell death, such as the starch granules and the protein bodies, it remains unclear to which extent their membrane systems remain intact. Should the membranes be preserved, they would have to be degraded by aleurone-derived lipases during germination to provide access of amylases and proteinases to the storage reserves. Indeed, lipases are produced in abundance by the aleurone, making this a conceivable scenario to be further investigated.

Due to their particular cell biology and the absence of cell corpse clearance, living and dead starchy endosperm cells have very similar appearance under the microscope and their particular mode of cell death has been termed cellular “mummification” (Doll and Ingram, 2022). During mummification, the densely packed starch granules and protein bodies may also serve as structural support, inhibiting the collapse of dead cells. In line with this notion, starch-defective mutants present a shrunken endosperm phenotype, indicating that the maintenance of cellular integrity is important for tissue- and ultimately seed -shape preservation (Bhave et al., 1990; Ma et al., 2014; Springer et al., 1986; K. Zhang et al., 2020).

Interestingly, the process of cell mummification is not specific to the cereal starchy endosperm, but has been also found in the perisperm (a maternally derived nucellus tissue serving an analogous function) of quinoa (María Paula López-Fernández and Maldonado, 2013). The presence of significant amounts of starch in both tissues opens the possibility that cells accumulating high levels of starch trigger this specific type of “preservative” cell death, possibly by generating a signaling cascade that trigger PCD in coordination with the completion of starch accumulation. However, other starchy storage tissues do not undergo cell death, for instance the potato tuber or the banana (Coleman, 2000; Maduwanthi and Marapana, 2019), suggesting that PCD is not an unavoidable consequence of starch storage. In cereal endosperm though, the close correlation between starch accumulation and cell death strongly suggests that these two processes are closely linked (Langer et al., 2023; Young et al., 1997).

### 3 Regulation of starchy endosperm cell death

The apparent developmental control of starchy endosperm cell death suggests an active regulation of this process. Although it has been associated with different molecular signalling pathways, mechanistic evidence on the molecular control of starchy endosperm cell death remains scarce.

Both ethylene and abscisic acid (ABA) have been implicated in starchy endosperm cell death. An excess of ethylene can trigger premature starchy endosperm cell death. As ethylene biosynthesis in the kernel peaks before starchy endosperm death, ethylene might be promoting this process, though further investigations are needed to support this hypothesis (Li et al., 2004; Young et al., 1997; Young and Gallie, 1999). By contrast, ABA negatively regulates starchy endosperm cell death: Blocking ABA signalling promotes starchy endosperm cell death, and as this is accompanied by an increase of ethylene levels, a cross-talk between these two hormones may coordinate the regulation of starchy endosperm cell death (Young and Gallie, 2000b). However, to date the mechanism that promotes endosperm cell death downstream of ethylene and ABA signalling remains unknown (Gallie and Young, 2004; Li et al., 2018). In particular, to our knowledge, no mutant with delayed endosperm cell death have been reported in mutants that are insensitive to ethylene, or defective in ethylene biosynthesis, raising the question whether the effect of hormonal treatment on cell death acceleration is a direct effect or rather an indirect consequence of ethylene treatment.

Starchy endosperm cell death has been shown to occur prematurely under excessive oxidative stress. Loss of DEK40 or DEK66 causes mitochondrial dysfunction and excessive production of reactive oxygen species (ROS), resulting in early starchy endosperm cell death in maize (Ren et al., 2019; Wei et al., 2023). Likewise, high ROS levels and precocious cell death have been detected in the starchy endosperm of *mads29* mutants in wheat, and of *flr4* and *flr14* mutant in rice (He et al., 2023; Liu et al., 2023). Interestingly, ROS production in rice endosperm is reduced by METALLOTHIONEIN 2b, which is stabilized by the scaffold protein WHITE CORE RATE 1 (WCR1). Downregulation of WRC1 results thus in excessive ROS production and premature starchy endosperm cell death (Wu et al., 2022). Besides ROS, also other sources of cellular stress can cause a premature starchy endosperm cell death. A mutation in *FLOURY4*, encoding for a 19kDa ZEIN, makes that the resulting protein is unable to detach from the plasma membrane, causes a endoplasmic reticulum- (ER-) stress and early endosperm cell death (Wang et al., 2014).

The level of endoreplication (replication of the nuclear genome without subsequent mitosis) is another cellular parameter that has been associated cell death execution, as cells with the highest ploidy levels are more prone to trigger cell death in barley (Nowicka et al., 2021). Interestingly, perturbation of the retinoblastoma (RBR) pathway in maize results in both increased levels of endoreduplication and cell death, though it remains unclear if RBR directly or indirectly influences cell death (Sabelli et al., 2013). Interestingly, loss of ATAXIA-TELANGIECTASIA MUTATED (ATM) and ATM AND RAD3-RELATED (ATR), which coordinate the DNA damage response, has been shown to not only cause accumulation of DNA damage, but also early endoreplication and premature cell death (Pedroza-Garcia et al., 2021).

The high susceptibility of endosperm cells to different cellular stresses may result from the formidable metabolic activity that is required during seed filling. Consequently, manipulating the genes and pathways listed above might generate a stressful state that eventually is only the last straw that breaks the camel's back. Thus, the actual gene network and the molecular mechanisms that prepare and execute cell death during regular development remain unclear. Whether core dPCD genes are involved, and which other proteins may be activated or inactivated to achieve the particular “preservative” outcome of endosperm cell death, remains an intriguing riddle to solve.

### 4 What is the function of starchy endosperm cell death?

The question of the biological function of starchy endosperm cell death remains subject to speculation, also due to the lack of mutants that specifically affect starchy endosperm cell death without disturbing other developmental processes. Starchy endosperm cell death happens also in wild species (Dermastia et al., 2009; Saada et al., 2021), suggesting that it is a result of natural rather than human selection. A death process that preserves nutrient-filled cell corpses might be a way to maximize nutrient storage by avoiding that stored nutrients are metabolized by the endosperm cells themselves. It is notable that starchy storage tissues composed of living cells, as are found for instance in banana or potatoes, have a much shorter lifespan than cereal endosperm and progressively consume the resources they store (Coleman, 2000; Maduwanthi and Marapana, 2019). Moreover, keeping the endosperm as a living tissue after desiccation would require substantial energy to rehydrate the cells and to produce the machinery responsible for nutrient remobilization. During hydration and subsequent germination, the surviving aleurone layer(s) secrete the lytic enzymes that remobilize the reserves stored in the dead starchy endosperm to fuel seedling growth. For this to happen efficiently, the starchy endosperm should to be devoid of major diffusion barriers to facilitate diffusion of lytic enzymes and mobilized nutrients (Sabelli and Larkins, 2009). In sum, by keeping only the aleurone layer(s) alive, cereals may save energy that would be consumed by a living starchy endosperm prior to desiccation and during germination. Identification of starchy endosperm cell death mechanisms and their targeted modulation will be crucial to test this hypothesis in the future.

Notably, defects in grain filling are often associated with premature starchy endosperm cell death. Shrunken endosperm (Wei et al., 2023; Young et al., 1997) or grain chalkiness (He et al., 2023; Wu et al., 2022) are observed in genetic backgrounds that also display an early starchy endosperm cell death, suggesting a connection between cell death timing and grain filling. As cell death effectively terminates endosperm filling, an early cell death might interfere with completion of grain filling, leading to suboptimal reserve compound deposition. Vice versa, defects in grain filling may trigger a precocious endosperm cell death. Thus, the interplay of grain filling and starchy endosperm cell death may be an interesting breeding target for improvement in grain quality.

## IV) Post-germination cell death of the surviving endosperm

In most angiosperms a part of the endosperm does not die and remains alive until germination. However, these surviving endosperm tissues will eventually die during or shortly after germination. Post-germination endosperm cell death has been intensively investigated in cereals and in Ricinus, but less in other species such as in Arabidopsis. In analogy to cereal aleurone cell death, some studies suggested that germination-induced changes in Arabidopsis aleurone cell morphology, most notably intense vacuolization (Bethke et al., 2007), lead to aleurone programmed cell death (Alonso-Peral et al., 2010). However, to our knowledge no investigation to date has provided conclusive evidence showing that Arabidopsis aleurone cells actually undergo a genetically controlled PCD during germination.

### 1 PCD in the cereal aleurone

As in Arabidopsis and many other taxa, the aleurone in cereals is composed of one or a few cell layers that survive throughout grain development and desiccation until germination. During germination, the cereal aleurone starts a maturation program that is eventually terminated by PCD (Zheng and Wang, 2014). Aleurone maturation allows the production and the secretion of enzymes, like alpha amylase, involved in the mobilization of starchy endosperm reserves. Aleurone storage lipids are also mobilized and the numerous storage vacuoles in the cytoplasm progressively merge into a large central lytic vacuole. Aleurone vacuolization is followed by programmed cell death and these two processes are tightly linked (Zheng et al., 2017) (Figure 2F-H). Aleurone maturation and PCD are promoted by the phytohormone gibberellic acid (GA) while being inhibited by its antagonist ABA (Kuo et al., 1996; Wang et al., 1996). GA is mainly produced by the germinating embryo, which is where aleurone maturation and PCD are first initiated before spreading outwards (Domínguez et al., 2004; Wang et al., 1996). Correct timing of PCD depends on GA signaling, involving the DELLA inhibitor protein SLN and the GAMyb transcription factor (Aoki et al., 2014; Guo and Ho, 2008). Bringing up the rear of a rapid cellular maturation process, aleurone PCD has been a convenient model system to study PCD regulation in cereals. In vitro cultivated aleurone cells, either as intact layers or as protoplasts, can be treated with GA to trigger PCD, which can be conveniently monitored by using dyes staining living cells such as fluorescein diacetate or staining dead cells such as propidium iodide (Kuo et al., 1996; Wang et al., 1996).

**Figure 3:**
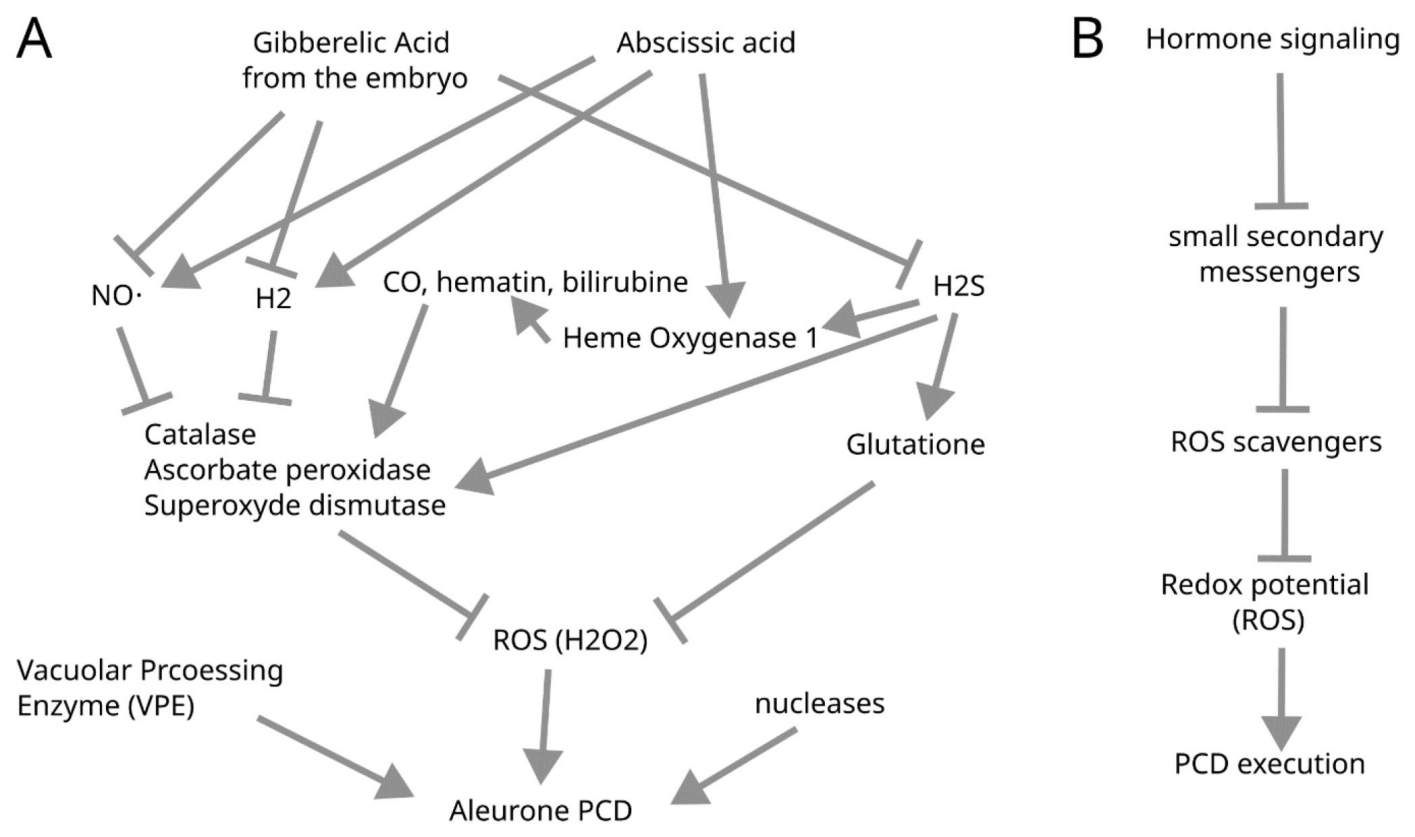
Regulation of aleurone PCD in cereals. A) Relationship between the molecular actors involved in PCD. B) Summary of the main processes involved in cereal PCD.

Apart from hormonal control, aleurone PCD is also regulated by the cellular redox potential. Higher ROS levels caused by high-light or application of hydrogen peroxide cause precocious vacuolization and PCD (Bethke and Jones, 2001; Wu et al., 2014, 2011). Furthermore, GA treatment causes a rapid ROS burst that lead to PCD later in the aleurone maturation process (Aoki et al., 2014). Conversely, antioxidants delay or inhibit aleurone PCD. Among them, a number of small molecules was shown to reduce the redox potential of aleurone cells, such as nitric oxide (NO) (Beligni et al., 2002; Bethke et al., 2004; Wu et al., 2015), hydrogen sulfide (H2S) (Xie et al., 2014; Zhang et al., 2015), hydrogen-rich water (Wu et al., 2021), CO, hematin or bilirubin, produced by heme oxygenase 1 (HO1) (Wu et al., 2011) (Figure 3A). These molecules regulate the expression and activity of antioxidant enzymes like superoxide dismutases, ascorbate peroxidases, catalases, peroxidases, or guaiacol peroxidases, which scavenge ROS and decrease thus the oxidation potential of aleurone cells. An elegant study dissecting the influence of the intra- and extracellular redox potential showed that only the intracellular redox potential increases following ROS treatment, suggesting an intracellular signaling downstream of ROS that promotes PCD execution (Mark et al., 2016). Collectively, these data suggest that the increased redox potential of aleurone cells eventually leading to PCD is caused by progressive inhibition of antioxidant molecules (NO, H2S, H2, HO1…) and ROS scavengers. The modulation of redox-related gene expression in turn is modulated epigenetically by the action of histone acetyltransferases (HAT) and deacetylases (HDAC) (Aoki et al., 2014; Hou et al., 2017). Whether an increase in ROS production occurs simultaneously to the global decrease in antioxidant levels remains unclear. Beta-oxidation of the storage lipids during nutrient mobilization has been proposed to be a major source of intracellular ROS prior PCD onset (Palma and Kermode, 2003).

In any case, aleurone cell death likely happens when a certain threshold in intracellular oxidation level is reached in mature cells. What happens downstream of ROS remains poorly understood. On the one hand different ROS can act as intra-and intercellular signaling molecules triggering a variety of processes (Mittler et al., 2022). On the other hand, high ROS levels have been proposed to act as toxins by oxidizing many molecules including membrane lipids, which may cause plasma membrane rupture (Fath et al., 2001). However, the identification of additional cellular activities suggests that ROS levels do not reach toxic levels in the early stages of PCD execution. For instance, cysteine protease activity increases during aleurone PCD (Fath et al., 2000), and inhibition of vacuolar processing enzymes (VPEs) delays cell death, indicating a contribution of VPE activity to PCD execution (Xiao et al., 2021; H. Zhang et al., 2020; Zheng et al., 2017). However, the exact mode of action of these proteases is not known. Aleurone PCD is also marked by increased nuclease activity, possibly contributing to the catabolism of nucleic acids (Bissenbaev et al., 2011; Domínguez et al., 2004; Fath et al., 2000, 1999). Overall, the execution of aleurone PCD downstream of ROS signals, as well as the postmortem clearance of the cell corpses remain still insufficiently understood.

### 2 PCD in the Ricinus endosperm

A different case of post-germination endosperm PCD has been described in castor bean (*Ricinus communis*). In castor bean, several layers of endosperm cells persist after germination, clinging to the emerging cotyledons. The endosperm thus covers and protects the young cotyledons during the first phase of seedling establishment. It has been shown that castor bean endosperm remobilizes its stored nutrients to fuel seedling growth. Like in the cereal aleurone, nutrient remobilization in castor bean endosperm is followed by PCD, with cells dying first in the vicinity of the embryo before cell death spreads to the outermost endosperm layers (Schmid et al., 1999). Around 6 days after germination and having transferred most of its oil and protein reserves to the embryo, the endosperm detaches from the expanding cotyledons (Schmid et al., 1999, 1998). Cell death progression correlates with the appearance of ER-derived vesicles called “ricinosomes”. Ricinosomes accumulate ER-retained KDEL-cysteine endopeptidases (KDEL-CysEPs) (Schmid et al., 1998) that are activated just before cell death execution (Schmid et al., 1999). In Arabidopsis, the orthologous KDEL-tailed CYSTEINE ENDOPEPTIDASE (CEP) genes are transcriptionally associated with PCD processes (Zhou et al., 2016), promote PCD in the tapetum and in the xylem in Arabidopsis, suggesting that KDEL-CysEPs might be involved in executing Ricinus endosperm cell death (Han et al., 2019; Hierl et al., 2012; Zhang et al., 2014). However, how the KDEL-CysEPs promote cell death remains unclear; they are thought to remodel the cell wall through the cleavage of hydroxyproline-rich glycoproteins (HRGPs), which form a part of the cell wall scaffold (Helm et al., 2008). While being a classical model for PCD, further studies on castor bean endosperm cell death would be necessary to understand the molecular mechanisms involved. Interestingly, GA-regulation of post-germination PCD in tomato endosperm suggests a conserved mechanism between cereal aleurone PCD and post-germination endosperm PCD in at least some other dicot taxa (DeBono and Greenwood, 2006).

### 3 Function of PCD post-germination?

Despite much is known on its regulation, the actual function of post-germination endosperm PCD remains enigmatic. It is tempting to speculate that the endosperm as an ultimately dispensable tissue can mobilize its own reserves, as well as produce and secrete enzymes, at extremely high rates, regardless of the consequences for its own survival. There lies a conceivable selective advantage in sacrificing the endosperm to maximize the fitness of the germinating seedling. As such germination-induced endosperm PCD would not fulfil a specific function *per se*, but rather be a willingly accepted consequence of extreme metabolic rates. In addition, it is possible that ultimately, cellular disintegration may maximize the release of nutrients and lytic enzymes via the loss of cellular integrity, though this is a hypothesis that would still need to be tested experimentally.

## V) Environmental influences on endosperm cell death

Most of the knowledge gained on developmentally regulated PCD are obtained in controlled lab conditions. However, outside the lab, plants are subjected to environmental factors that affect its development, and also influence dPCD processes. However, only few studies documented in how far environmental influences affect endosperm PCD: Analysis of starchy endosperm cell death in winter wheat showed that nuclear deformation, mitochondria degeneration, plasma membrane permeation, and DNA laddering occur earlier under drought stress compared to control conditions (Li et al., 2018). In the post-germination aleurone, drought stress promotes vacuolization and PCD, in concert with a promotion of seed germination under these conditions (Wu et al., 2015). A significant increase in endogenous aleurone H2O2 levels has been measured under drought conditions, indicating increased oxidative stress. This may be, at least partially caused, by a decrease in the expression of the ROS scavenger -producing enzyme HO1. As in both starchy endosperm and aleurone, the entrance into cell death is sensitive to the redox state, the oxidative stress generated by the drought stress might trigger the accelerated cell death in both tissues (Wang et al., 2018). Surprisingly, under opposite water stress, i.e. waterlogging, the starchy endosperm cell death in wheat is also accelerated (Fan et al., 2013). However, waterlogging causes similar increase in oxidative stress (Cheng et al., 2016), and might thus work via a similar signaling pathway. These findings are in line with the general notion that starchy endosperm cells are highly sensitive to stresses, and that many perturbations can accelerate the onset of cell death. Considering the importance of endosperm cell death in relevant crops, a significant effort should be made to better understand the impact of environmental stresses on PCD, especially in the light of the increasing effects of climate change on crop yield stability.

## VI) Conclusions

Endosperm is a specialized transient organ in angiosperms and is consequently destined to die during seed development and germination. However, rather passively degenerating, endosperm cells execute diverse actively controlled programmed cell death processes at specific moments in seed development (Table 1). Depending on the different functional contexts, there are many ways for an endosperm cell to die. Degeneration of the unfertilized central cell in endosperm-less species is a way to avoid endosperm formation. Endosperm elimination around the embryo allows for optimal growth and development of the embryo while transferring endosperm-derived nutrients to the embryo. In cereals, the huge nutrient-packed starchy endosperm cells undergo a particular cell death process that conserves the stored nutrients and facilitates their remobilization during germination. Finally, the endosperm layers that survive throughout seed development eventually die during germination while releasing enzymes and their own storage reserves.

The different ways to execute cell death in different tissues or species suggest that various molecular mechanisms are involved (Table 1). Endosperm cell elimination in Arabidopsis is under the control of the ZOU and the NAC pathways, while starchy endosperm and aleurone cell death in cereal are highly sensitive to oxidative stress. In all cases, the precise genes involved in the execution of cell death remain poorly understood. Many genes belonging to gene or protein families involved in cell death execution in other contexts have been shown to be expressed in association with endosperm cell death. However, for most of them, their precise role in endosperm cell death execution remains speculative. While the hidden position of the endosperm within the seed does not facilitate functional studies, the investigation of endosperm PCD will be a key to understand this crucial process.

Due to the central role of the endosperm in agriculture, endosperm PCD is a topic of particularly high interest for plant breeding. Defects in endosperm PCD are associated with many unfavorable agronomic traits, indicating that a correct execution of PCD in the endosperm is critical to produce high-quality grain. Under environmental stresses, the timing of endosperm cell death is affected, which may lead to significant decrease in grain yield and quality. Consequently, future studies on endosperm cell death regulation and the impact of environmental factors will be necessary to anticipate the threat posed by climate change for agriculture.

## Figures and Tables

**Table 1:** Summary of the PCD process occurring during central cell and endosperm development in angiosperms.

PCD process	species	localization	Developmental timing	Known molecular regulators
Central cell degeneration	Podostomaceae, Orchids (?)	Embryo sac	Before fertilization	none
Endosperm elimination	In most angiosperms	Embryo surrounding region	During embryo invasive growth (often during mid and late seed development)	AtZOU, AtICE1 AtANAC087, AtANAC046, AtORE1, AtKIR1
Starchy endosperm conservative cell death	In cereals	Central endosperm	During the filling stage (late grain development)	Ethylene, ABA, ROS, ZmDEK40, ZmDEK66, TaMADS29, OsFLR4, OsFLR14, OsWCR1, ZmATR, ZmATM, ZmRBRs,
Post germination cell death	In endospermic species	Surviving mature endosperm	During and after germination	KDEL-CysEPs (?)
Aleurone cell death	In cereals	Outermost layer(s) of endosperm	During germination	ROS, GA, OsGAMyb, OsSLN, ABA, NO, H2S, HO1, hydrogen rich water, ROS and ROS scavenging enzymes (catalase, SOD, ascorbate peroxidases…), HAT, HDAC, OsVPEs,

## Highlights

This review discusses the current understanding of the cellular features, molecular regulation, and functional roles of the diverse endosperm cell death processes occurring across the angiosperm phylum.

## References

[R1] Alonso-Peral MM, Li J, Li Y, Allen RS, Schnippenkoetter W, Ohms S, White RG, Millar AA (2010). The microRNA159-regulated GAMYB-like genes inhibit growth and promote programmed cell death in Arabidopsis. Plant Physiology.

[R2] Aoki N, Ishibashi Y, Kai K, Tomokiyo R, Yuasa T, Iwaya-Inoue M (2014). Programmed cell death in barley aleurone cells is not directly stimulated by reactive oxygen species produced in response to gibberellin. Journal of Plant Physiology.

[R3] Arditti J (1992). Fundamentals of orchid biology / Joseph Arditti.

[R4] Arditti J, Ghani AKA (2000). Tansley Review No.110.: Numerical and physical properties of orchid seeds and their biological implications. The New Phytologist.

[R5] Baroux C, Spillane C, Grossniklaus U (2002). Evolutionary origins of the endosperm in flowering plants. Genome Biology.

[R6] Beligni MV, Fath A, Bethke PC, Lamattina L, Jones RL (2002). Nitric oxide acts as an antioxidant and delays programmed cell death in barley aleurone layers. Plant Physiology.

[R7] Bethke PC, Badger MR, Jones RL (2004). Apoplastic synthesis of nitric oxide by plant tissues. The Plant Cell.

[R8] Bethke PC, Jones RL (2001). Cell death of barley aleurone protoplasts is mediated by reactive oxygen species. The Plant Journal: For Cell and Molecular Biology.

[R9] Bethke PC, Libourel IGL, Aoyama N, Chung Y-Y, Still DW, Jones RL (2007). The Arabidopsis aleurone layer responds to nitric oxide, gibberellin, and abscisic acid and is sufficient and necessary for seed dormancy. Plant Physiology.

[R10] Bhave MR, Lawrence S, Barton C, Hannah LC (1990). Identification and molecular characterization of shrunken-2 cDNA clones of maize. The Plant Cell.

[R11] Bissenbaev AK, Ishchenko AA, Taipakova SM, Saparbaev MK (2011). Presence of base excision repair enzymes in the wheat aleurone and their activation in cells undergoing programmed cell death. Plant Physiology and Biochemistry: PPB.

[R12] Boisnard-Lorig C, Colon-Carmona A, Bauch M, Hodge S, Doerner P, Bancharel E, Dumas C, Haseloff J, Berger F (2001). Dynamic Analyses of the Expression of the HISTONE::YFP Fusion Protein in Arabidopsis Show That Syncytial Endosperm Is Divided in Mitotic Domains. The Plant Cell.

[R13] Briggs CL (1993). Endosperm Development in Solanum nigrum L. Formation of the Zone of Separation and Secretion. Annals of Botany.

[R14] Briggs CL (1996). An Ultrastructural Study of The Embryo/Endosperm Interface in the Developing Seeds of Solanum nigrum L. Zygote to Mid Torpedo Stage. Annals of Botany.

[R15] Brown RC, Lemmon BE, Nguyen H, Olsen O-A (1999). Development of endosperm in Arabidopsis thaliana. Sexual Plant Reproduction.

[R16] Cannon MC, Terneus K, Hall Q, Tan L, Wang Y, Wegenhart BL, Chen L, Lamport DTA, Chen Y, Kieliszewski MJ (2008). Self-assembly of the plant cell wall requires an extensin scaffold. Proceedings of the National Academy of Sciences of the United States of America.

[R17] Chen Y, Ye D, Held MA, Cannon MC, Ray T, Saha P, Frye AN, Mort AJ, Kieliszewski MJ (2015). Identification of the Abundant Hydroxyproline-Rich Glycoproteins in the Root Walls of Wild-Type Arabidopsis, an ext3 Mutant Line, and Its Phenotypic Revertant. Plants (Basel, Switzerland).

[R18] Cheng X-X, Yu M, Zhang N, Zhou Z-Q, Xu Q-T, Mei F-Z, Qu L-H (2016). Reactive oxygen species regulate programmed cell death progress of endosperm in winter wheat (Triticum aestivum L.) under waterlogging. Protoplasma.

[R19] Coleman WK (2000). Physiological ageing of potato tubers: A Review. Annals of Applied Biology.

[R20] Cubría-Radío M, Nowack MK (2019). Transcriptional networks orchestrating programmed cell death during plant development. Current Topics in Developmental Biology.

[R21] Daneva A, Gao Z, Van Durme M, Nowack MK (2016). Functions and Regulation of Programmed Cell Death in Plant Development. Annual Review of Cell and Developmental Biology.

[R22] DeBono AG, Greenwood JS (2006). Characterization of programmed cell death in the endosperm cells of tomato seed: Two distinct death programs. Canadian Journal of Botany.

[R23] Denay G, Creff A, Moussu S, Wagnon P, Thévenin J, Gérentes M-F, Chambrier P, Dubreucq B, Ingram G (2014). Endosperm breakdown in Arabidopsis requires heterodimers of the basic helix-loop-helix proteins ZHOUPI and INDUCER OF CBP EXPRESSION 1. Development (Cambridge, England).

[R24] Dermastia M, Kladnik A, Koce Dolenc, Chourey PS (2009). A cellular study of teosinte Zea mays subsp. Parviglumis (Poaceae) caryopsis development showing several processes conserved in maize. American Journal of Botany.

[R25] Doll NM, Bovio S, Gaiti A, Marsollier A-C, Chamot S, Moussu S, Widiez T, Ingram G (2020). The Endosperm-Derived Embryo Sheath Is an Anti-adhesive Structure that Facilitates Cotyledon Emergence during Germination in Arabidopsis. Current Biology: CB.

[R26] Doll NM, Ingram GC (2022). Embryo-Endosperm Interactions. Annual Review of Plant Biology.

[R27] Doll NM, Just J, Brunaud V, Caïus J, Grimault A, Depège-Fargeix N, Esteban E, Pasha A, Provart NJ, Ingram GC, Rogowsky PM (2020). Transcriptomics at Maize Embryo/Endosperm Interfaces Identifies a Transcriptionally Distinct Endosperm Subdomain Adjacent to the Embryo Scutellum. The Plant Cell.

[R28] Doll NM, Van Hautegem T, Schilling N, De Rycke R, De Winter F, Fendrych M, Nowack MK (2023). Endosperm cell death promoted by NAC transcription factors facilitates embryo invasion in Arabidopsis. Current Biology: CB.

[R29] Domínguez F, Cejudo FJ (2014). Programmed cell death (PCD) : An essential process of cereal seed development and germination. Frontiers in Plant Science.

[R30] Domínguez F, Moreno J, Cejudo FJ (2004). A gibberellin-induced nuclease is localized in the nucleus of wheat aleurone cells undergoing programmed cell death. The Journal of Biological Chemistry.

[R31] Dumas C, Rogowsky P (2008). Fertilization and early seed formation. Comptes Rendus Biologies.

[R32] Eriksson O, Kainulainen K (2011). The evolutionary ecology of dust seeds. Perspectives in Plant Ecology, Evolution and Systematics.

[R33] Fan H-Y, Zhou Z-Q, Yang C-N, Jiang Z, Li J-T, Cheng X-X, Guo Y-J (2013). Effects of waterlogging on amyloplasts and programmed cell death in endosperm cells of Triticum aestivum L. Protoplasma.

[R34] Fath A, Bethke PC, Belligni MV, Spiegel YN, Jones RL (2001). Signalling in the cereal aleurone : Hormones, reactive oxygen and cell death. The New Phytologist.

[R35] Fath A, Bethke PC, Jones RL (1999). Barley aleurone cell death is not apoptotic : Characterization of nuclease activities and DNA degradation. The Plant Journal : For Cell and Molecular Biology.

[R36] Fath A, Bethke P, Lonsdale J, Meza-Romero R, Jones R (2000). Programmed cell death in cereal aleurone. Plant Molecular Biology.

[R37] Fourquin C, Beauzamy L, Chamot S, Creff A, Goodrich J, Boudaoud A, Ingram G (2016). Mechanical stress mediated by both endosperm softening and embryo growth underlies endosperm elimination in Arabidopsis seeds. Development (Cambridge, England).

[R38] Friedman WE (1998). The evolution of double fertilization and endosperm : An ”historical” perspective. Sexual Plant Reproduction.

[R39] Gallie DR, Young TE (2004). The ethylene biosynthetic and perception machinery is differentially expressed during endosperm and embryo development in maize. Molecular Genetics and Genomics : MGG.

[R40] Golovina EA, Hoekstra FA, Aelst van AC (2000). Programmed cell death or desiccation tolerance : Two possible routes for wheat endosperm cells. Seed Science Research.

[R41] Guo W-J, Ho T-H (2008). An abscisic acid-induced protein, HVA22, inhibits gibberellin-mediated programmed cell death in cereal aleurone cells. Plant Physiology.

[R42] Han J, Li H, Yin B, Zhang Y, Liu Y, Cheng Z, Liu D, Lu H (2019). The papain-like cysteine protease CEP1 is involved in programmed cell death and secondary wall thickening during xylem development in Arabidopsis. Journal of Experimental Botany.

[R43] He W, Li W, Luo X, Tang Y, Wang L, Yu F, Lin Q (2023). Rice FERONIA-LIKE RECEPTOR 3 and 14 affect grain quality by regulating redox homeostasis during endosperm development. Journal of Experimental Botany.

[R44] Helm M, Schmid M, Hierl G, Terneus K, Tan L, Lottspeich F, Kieliszewski MJ, Gietl C (2008). KDEL-tailed cysteine endopeptidases involved in programmed cell death, intercalation of new cells, and dismantling of extensin scaffolds. American Journal of Botany.

[R45] Hierl G, Vothknecht U, Gietl C (2012). Programmed cell death in Ricinus and Arabidopsis : The function of KDEL cysteine peptidases in development. Physiologia Plantarum.

[R46] Hofmann K (2020). The Evolutionary Origins of Programmed Cell Death Signaling. Cold Spring Harbor Perspectives in Biology.

[R47] Hou H, Zheng X, Zhang H, Yue M, Hu Y, Zhou H, Wang Q, Xie C, Wang P, Li L (2017). Histone Deacetylase Is Required for GA-Induced Programmed Cell Death in Maize Aleurone Layers. Plant Physiology.

[R48] Ingram GC (2020). Family plot : The impact of the endosperm and other extra-embryonic seed tissues on angiosperm zygotic embryogenesis. F1000Research.

[R49] Ishimaru T, Parween S, Saito Y, Masumura T, Kondo M, Sreenivasulu N (2022). Laser microdissection transcriptome data derived gene regulatory networks of developing rice endosperm revealed tissue-and stage-specific regulators modulating starch metabolism. Plant Molecular Biology.

[R50] Jiménez-Durán K, Pérez-Pacheco MK, Wong R, Collazo-Ortega M, Márquez-Guzmán J (2021). Programmed cell death is the cause of central cell degeneration and single fertilization in Marathrum schiedeanum (Cham.) Tul (Podostemaceae. Aquatic Botany.

[R51] Johansson M, Walles B (1994). Functional Anatomy of the Ovule in Broad Bean (Vicia faba L.) : Ultrastructural Seed Development and Nutrient Pathways. Annals of Botany.

[R52] Johri BM (1984). Embryology of Angiosperms.

[R53] Johri BM, Ambegaokar KB, Srivastava PS (1992). Comparative Embryology of Angiosperms.

[R54] Kanaoka MM, Pillitteri LJ, Fujii H, Yoshida Y, Bogenschutz NL, Takabayashi J, Zhu J-K, Torii KU (2008). SCREAM/ICE1 and SCREAM2 specify three cell-state transitional steps leading to arabidopsis stomatal differentiation. The Plant Cell.

[R55] Khoo U, Wolf MJ (1970). Origin and Development of Protein Granules in Maize Endosperm. American Journal of Botany.

[R56] Kobayashi H, Ikeda TM, Nagata K (2013). Spatial and temporal progress of programmed cell death in the developing starchy endosperm of rice. Planta.

[R57] Kodahl N, Johansen BB, Rasmussen FN (2015). The embryo sac of Vanilla imperialis (Orchidaceae) is six-nucleate, and double fertilization and formation of endosperm are not observed. Botanical Journal of the Linnean Society.

[R58] Kuo A, Cappelluti S, Cervantes-Cervantes M, Rodriguez M, Bush DS (1996). Okadaic acid, a protein phosphatase inhibitor, blocks calcium changes, gene expression, and cell death induced by gibberellin in wheat aleurone cells. The Plant Cell.

[R59] Langer M, Hilo A, Guan J-C, Koch KE, Xiao H, Verboven P, Gündel A, Wagner S, Ortleb S, Radchuk V, Mayer S (2023). Causes and consequences of endogenous hypoxia on growth and metabolism of developing maize kernels. Plant Physiology.

[R60] Li C, Li C, Wang B, Zhang R, Fu K, Gale WJ, Li C (2018). Programmed cell death in wheat (Triticum aestivum L.) endosperm cells is affected by drought stress. Protoplasma.

[R61] Li R, Lan S-Y, Xu Z-X (2004). Programmed cell death in wheat during starchy endosperm development. Zhi Wu Sheng Li Yu Fen Zi Sheng Wu Xue Xue Bao = Journal of Plant Physiology and Molecular Biology.

[R62] Liu G, Zhang R, Li S, Ullah R, Yang F, Wang Z, Guo W, You M, Li B, Xie C, Wang L (2023). TaMADS29 interacts with TaNF-YB1 to synergistically regulate early grain development in bread wheat. Science China Life Sciences.

[R63] Locato V, De Gara L (2018). Programmed Cell Death in Plants : An Overview. Methods in Molecular Biology (Clifton, NJ).

[R64] Lombardi L, Ceccarelli N, Picciarelli P, Lorenzi R (2007). DNA degradation during programmed cell death in Phaseolus coccineus suspensor. Plant Physiology and Biochemistry : PPB.

[R65] López-Fernández MP, Maldonado S (2013a). Programmed cell death during quinoa perisperm development. Journal of Experimental Botany.

[R66] López-Fernández MP, Maldonado S (2013b). Ricinosomes provide an early indicator of suspensor and endosperm cells destined to die during late seed development in quinoa (Chenopodium quinoa). Annals of Botany.

[R67] Leleeka Devi M, Tandon Sanavar R, Uniya PL (2016). Features of seeds of Podostemaceae and their survival strategy in freshwater ecosystems.

[R68] Ma J, Jiang QT, Wei L, Wang JR, Chen GY, Liu YX, Li W, Wei YM, Liu C, Zheng YL (2014). Characterization of shrunken endosperm mutants in barley. Gene.

[R69] Maduwanthi SDT, Marapana RaUJ (2019). Induced Ripening Agents and Their Effect on Fruit Quality of Banana. International Journal of Food Science.

[R70] Maekawa T, Kashkar H, Coll NS (2023). Dying in self-defence : A comparative overview of immunogenic cell death signalling in animals and plants. Cell Death and Differentiation.

[R71] Mansfield SG, Briarty LG (1991). Early embryogenesis in Arabidopsis thaliana. II. The developing embryo. Canadian Journal of Botany.

[R72] Mark C, Zór K, Heiskanen A, Dufva M, Emnéus J, Finnie C (2016). Monitoring intra-and extracellular redox capacity of intact barley aleurone layers responding to phytohormones. Analytical Biochemistry.

[R73] Marsollier A-C, Ingram G (2018). Getting physical : Invasive growth events during plant development. Current Opinion in Plant Biology.

[R74] Minina EA, Dauphinee AN, Ballhaus F, Gogvadze V, Smertenko AP, Bozhkov PV (2021). Apoptosis is not conserved in plants as revealed by critical examination of a model for plant apoptosis-like cell death. BMC Biology.

[R75] Mittler R, Zandalinas SI, Fichman Y, Van Breusegem F (2022). Reactive oxygen species signalling in plant stress responses. Nature Reviews Molecular Cell Biology.

[R76] Moose SP, Dudley JW, Rocheford TR (2004). Maize selection passes the century mark : A unique resource for 21st century genomics. Trends in Plant Science.

[R77] Moussu S, Doll NM, Chamot S, Brocard L, Creff A, Fourquin C, Widiez T, Nimchuk ZL, Ingram G (2017). ZHOUPI and KERBEROS Mediate Embryo/Endosperm Separation by Promoting the Formation of an Extracuticular Sheath at the Embryo Surface. The Plant Cell.

[R78] Nagasawa N, Hibara K, Heppard EP, Vander Velden KA, Luck S, Beatty M, Nagato Y, Sakai H (2013). GIANT EMBRYO encodes CYP78A13, required for proper size balance between embryo and endosperm in rice. The Plant Journal : For Cell and Molecular Biology.

[R79] Nowicka A, Kovacik M, Tokarz B, Vrána J, Zhang Y, Weigt D, Doležel J, Pecinka A (2021). Dynamics of endoreduplication in developing barley seeds. Journal of Experimental Botany.

[R80] Olsen O-A (2001). ENDOSPERM DEVELOPMENT : Cellularization and Cell Fate Specification. Annual Review of Plant Physiology and Plant Molecular Biology.

[R81] Paes de Melo B, Carpinetti de PA, Fraga OT, Rodrigues-Silva PL, Fioresi VS, de Camargos LF, Ferreira da MFS (2022). Abiotic Stresses in Plants and Their Markers : A Practice View of Plant Stress Responses and Programmed Cell Death Mechanisms. Plants (Basel, Switzerland).

[R82] Palma K, Kermode AR (2003). Metabolism of hydrogen peroxide during reserve mobilization and programmed cell death of barley (Hordeum vulgare L.) aleurone layer cells. Free Radical Biology & Medicine.

[R83] Pedroza-Garcia JA, Eekhout T, Achon I, Nisa M-U, Coussens G, Vercauteren I, Van den Daele H, Pauwels L, Van Lijsebettens M, Raynaud C, De Veylder L (2021). Maize ATR safeguards genome stability during kernel development to prevent early endosperm endocycle onset and cell death. The Plant Cell.

[R84] Philbrick CT, Alejandro Novelo R (1997). Ovule number, seed number and seed size in Mexican and North American species of Podostemaceae. Aquatic Botany.

[R85] Raghavan V (2003). Some reflections on double fertilization, from its discovery to the present. The New Phytologist.

[R86] Ren RC, Lu X, Zhao YJ, Wei YM, Wang LL, Zhang L, Zhang WT, Zhang C, Zhang XS, Zhao XY (2019). Pentatricopeptide repeat protein DEK40 is required for mitochondrial function and kernel development in maize. Journal of Experimental Botany.

[R87] Rutishauser R (1997). Structural and developmental diversity in Podostemaceae (river-weeds. Aquatic Botany.

[R88] Saada S, Solomon CU, Drea S (2021). Programmed Cell Death in Developing Brachypodium distachyon Grain. International Journal of Molecular Sciences.

[R89] Sabelli PA (2012). Replicate and die for your own good : Endoreduplication and cell death in the cereal endosperm. Journal of Cereal Science.

[R90] Sabelli PA, Larkins BA (2009). The development of endosperm in grasses. Plant Physiology.

[R91] Sabelli PA, Liu Y, Dante RA, Lizarraga LE, Nguyen HN, Brown SW, Klingler JP, Yu J, LaBrant E, Layton TM, Feldman M (2013). Control of cell proliferation, endoreduplication, cell size, and cell death by the retinoblastoma-related pathway in maize endosperm. Proceedings of the National Academy of Sciences of the United States of America.

[R92] Schmid M, Simpson D, Gietl C (1999). Programmed cell death in castor bean endosperm is associated with the accumulation and release of a cysteine endopeptidase from ricinosomes. Proceedings of the National Academy of Sciences of the United States of America.

[R93] Schmid M, Simpson D, Kalousek F, Gietl C (1998). A cysteine endopeptidase with a C-terminal KDEL motif isolated from castor bean endosperm is a marker enzyme for the ricinosome, a putative lytic compartment. Planta.

[R94] Sehgal A, Khurana JP, Sethi M, Ara H (2011). Occurrence of unique three-celled megagametophyte and single fertilization in an aquatic angiosperm-Dalzellia zeylanica (Podostemaceae-Tristichoideae). Sexual Plant Reproduction.

[R95] Sehgal A, Mann N, Mohan Ram HY (2014). Structural and developmental variability in the female gametophyte of Griffithella hookeriana, Polypleurum stylosum, and Zeylanidium lichenoides and its bearing on the occurrence of single fertilization in Podostemaceae. Plant Reproduction.

[R96] Springer B, Werr W, Starlinger P, Bennett DC, Zokolica M, Freeling M (1986). The Shrunken gene on chromosome 9 of Zea mays L is expressed in various plant tissues and encodes an anaerobic protein. Molecular & General Genetics : MGG.

[R97] Terasaka O, Niitsu T, Tanaka R (1979). Single fertilization inSpiranthes sinensis. The Botanical Magazine Tokyo.

[R98] Van Hautegem T, Waters AJ, Goodrich J, Nowack MK (2015). Only in dying, life : Programmed cell death during plant development. Trends in Plant Science.

[R99] Wang G, Qi W, Wu Q, Yao D, Zhang J, Zhu J, Wang G, Wang G, Tang Y, Song R (2014). Identification and Characterization of Maize floury4 as a Novel Semidominant Opaque Mutant That Disrupts Protein Body Assembly. Plant Physiology.

[R100] Wang G, Xiao Y, Deng X, Zhang H, Li T, Chen H (2018). Exogenous Hydrogen Peroxide Contributes to Heme Oxygenase-1 Delaying Programmed Cell Death in Isolated Aleurone Layers of Rice Subjected to Drought Stress in a cGMP-Dependent Manner. Frontiers in Plant Science.

[R101] Wang M, Oppedijk BJ, Lu X, Van Duijn B, Schilperoort RA (1996). Apoptosis in barley aleurone during germination and its inhibition by abscisic acid. Plant Molecular Biology.

[R102] Wegel E, Vallejos RH, Christou P, Stöger E, Shaw P (2005). Large-scale chromatin decondensation induced in a developmentally activated transgene locus. Journal of Cell Science.

[R103] Wei YM, Wang BH, Shao DJ, Yan RY, Wu JW, Zheng GM, Zhao YJ, Zhang XS, Zhao XY (2023). Defective kernel 66 encodes a GTPase essential for kernel development in maize. Journal of Experimental Botany.

[R104] Wojciechowska M, Olszewska MJ (2003). Endosperm degradation during seed development of Echinocystis lobata (Cucurbitaceae) as a manifestation of programmed cell death (PCD) in plants. Folia Histochemica Et Cytobiologica.

[R105] Wu B, Yun P, Zhou H, Xia D, Gu Y, Li P, Yao J, Zhou Z, Chen J, Liu R, Cheng S (2022). Natural variation in WHITE-CORE RATE 1 regulates redox homeostasis in rice endosperm to affect grain quality. The Plant Cell.

[R106] Wu H, Zheng Y, Liu J, Zhang H, Chen H (2015). Heme Oxygenase-1 Delays Gibberellin-Induced Programmed Cell Death of Rice Aleurone Layers Subjected to Drought Stress by Interacting with Nitric Oxide. Frontiers in Plant Science.

[R107] Wu M, Huang J, Xu S, Ling T, Xie Y, Shen W (2011). Haem oxygenase delays programmed cell death in wheat aleurone layers by modulation of hydrogen peroxide metabolism. Journal of Experimental Botany.

[R108] Wu M, Li J, Wang F, Li F, Yang J, Shen W (2014). Cobalt alleviates GA-induced programmed cell death in wheat aleurone layers via the regulation of H2O2 production and heme oxygenase-1 expression. International Journal of Molecular Sciences.

[R109] Wu M, Xie X, Wang Z, Zhang J, Luo Z, Shen W, Yang J (2021). Hydrogen-rich water alleviates programmed cell death induced by GA in wheat aleurone layers by modulation of reactive oxygen species metabolism. Plant Physiology and Biochemistry : PPB.

[R110] Xiao Y, Zhang L, Zhang H, Feng H, Li Z, Chen H (2021). Interaction between endogenous H2O2 and OsVPE3 in the GA-induced PCD of rice aleurone layers. Plant Cell Reports.

[R111] Xie Y, Zhang C, Lai D, Sun Y, Samma MK, Zhang J, Shen W (2014). Hydrogen sulfide delays GA-triggered programmed cell death in wheat aleurone layers by the modulation of glutathione homeostasis and heme oxygenase-1 expression. Journal of Plant Physiology.

[R112] Xiong H, Wang W, Sun M-X (2021). Endosperm development is an autonomously programmed process independent of embryogenesis. The Plant Cell.

[R113] Yang S, Johnston N, Talideh E, Mitchell S, Jeffree C, Goodrich J, Ingram G (2008). The endosperm-specific ZHOUPI gene of Arabidopsis thaliana regulates endosperm breakdown and embryonic epidermal development. Development (Cambridge, England).

[R114] Yang W, Gao M, Yin X, Liu J, Xu Y, Zeng L, Li Q, Zhang S, Wang J, Zhang X, He Z (2013). Control of rice embryo development, shoot apical meristem maintenance, and grain yield by a novel cytochrome p450. Molecular Plant.

[R115] Yeung EC (2017). A perspective on orchid seed and protocorm development. Botanical Studies.

[R116] Young TE, Gallie DR (1999). Analysis of programmed cell death in wheat endosperm reveals differences in endosperm development between cereals. Plant Molecular Biology.

[R117] Young TE, Gallie DR (2000a). Programmed cell death during endosperm development. Plant Molecular Biology.

[R118] Young TE, Gallie DR (2000b). Regulation of programmed cell death in maize endosperm by abscisic acid. Plant Molecular Biology.

[R119] Young TE, Gallie DR, DeMason DA (1997). Ethylene-Mediated Programmed Cell Death during Maize Endosperm Development of Wild-Type and shrunken2 Genotypes. Plant Physiology.

[R120] Zhang D, Liu D, Lv X, Wang Y, Xun Z, Liu Z, Li F, Lu H (2014). The Cysteine Protease CEP1, a Key Executor Involved in Tapetal Programmed Cell Death, Regulates Pollen Development in Arabidopsis[W][OPEN]. The Plant Cell.

[R121] Zhang H, Xiao Y, Deng X, Feng H, Li Z, Zhang L, Chen H (2020). OsVPE3 Mediates GA-induced Programmed Cell Death in Rice Aleurone Layers via Interacting with Actin Microfilaments. Rice (New York, NY).

[R122] Zhang K, Guo L, Cheng W, Liu B, Li W, Wang F, Xu C, Zhao X, Ding Z, Zhang K, Li K (2020). SH1-dependent maize seed development and starch synthesis via modulating carbohydrate flow and osmotic potential balance. BMC Plant Biology.

[R123] Zhang P, Allen WB, Nagasawa N, Ching AS, Heppard EP, Li H, Hao X, Li X, Yang X, Yan J, Nagato Y (2012). A transposable element insertion within ZmGE2 gene is associated with increase in embryo to endosperm ratio in maize. TAG Theoretical and Applied Genetics Theoretische Und Angewandte Genetik.

[R124] Zhang Y-X, Hu K-D, Lv K, Li Y-H, Hu L-Y, Zhang X-Q, Ruan L, Liu Y-S, Zhang H (2015). The Hydrogen Sulfide Donor NaHS Delays Programmed Cell Death in Barley Aleurone Layers by Acting as an Antioxidant. Oxidative Medicine and Cellular Longevity.

[R125] Zheng Y, Wang Z (2014). Differentiation mechanism and function of the cereal aleurone cells and hormone effects on them. Plant Cell Reports.

[R126] Zheng Y, Wang Z (2015). The cereal starch endosperm development and its relationship with other endosperm tissues and embryo. Protoplasma.

[R127] Zheng Y, Zhang H, Deng X, Liu J, Chen H (2017). The relationship between vacuolation and initiation of PCD in rice (Oryza sativa) aleurone cells. Scientific Reports.

[R128] Zhou L-Z, Höwing T, Müller B, Hammes UZ, Gietl C, Dresselhaus T (2016). Expression analysis of KDEL-CysEPs programmed cell death markers during reproduction in Arabidopsis. Plant Reproduction.

